# Apoptin as a Tumor-Specific Therapeutic Agent: Current Perspective on Mechanism of Action and Delivery Systems

**DOI:** 10.3389/fcell.2020.00524

**Published:** 2020-06-25

**Authors:** Waseem Akram Malla, Richa Arora, Raja Ishaq Nabi Khan, Sonalika Mahajan, Ashok Kumar Tiwari

**Affiliations:** ^1^Division of Veterinary Biotechnology, ICAR-Indian Veterinary Research Institute, Izatnagar, India; ^2^Division of Biological Standardisation, ICAR-Indian Veterinary Research Institute, Izatnagar, India

**Keywords:** apoptin, oncotherapy, oncolytic viral genes, anticancer genes, cancer gene therapy, chicken infectious anemia, CAV, VP3

## Abstract

Cancer remains one of the leading causes of death worldwide in humans and animals. Conventional treatment regimens often fail to produce the desired outcome due to disturbances in cell physiology that arise during the process of transformation. Additionally, development of treatment regimens with no or minimum side-effects is one of the thrust areas of modern cancer research. Oncolytic viral gene therapy employs certain viral genes which on ectopic expression find and selectively destroy malignant cells, thereby achieving tumor cell death without harming the normal cells in the neighborhood. Apoptin, encoded by Chicken Infectious Anemia Virus’ VP3 gene, is a proline-rich protein capable of inducing apoptosis in cancer cells in a selective manner. In normal cells, the filamentous Apoptin becomes aggregated toward the cell margins, but is eventually degraded by proteasomes without harming the cells. In malignant cells, after activation by phosphorylation by a cancer cell-specific kinase whose identity is disputed, Apoptin accumulates in the nucleus, undergoes aggregation to form multimers, and prevents the dividing cancer cells from repairing their DNA lesions, thereby forcing them to undergo apoptosis. In this review, we discuss the present knowledge about the structure of Apoptin protein, elaborate on its mechanism of action, and summarize various strategies that have been used to deliver it as an anticancer drug in various cancer models.

## Introduction

Despite tremendous advances in medical sciences, molecular biology, and cancer biology, cancer continues to remain one of the leading causes of death worldwide just next to heart diseases. Of all the 16 million cancer patients reported per year, more than 70% of the cases are reported from low- and middle-income countries (Jemal et al., 2011; WHO, 2018; Siegel et al., 2019). Not just humans, various types of cancer continue to affect the health and wellbeing of all the animal species (CancerQuest, 2018; American Veterinary Medical Association, 2019). Cancer cells differ from normal cells with respect to mutations in critical genes responsible for cell division, cell survival, and cell death. Proto-oncogenes are typically activated, tumor suppressor genes like p53, PTEN, etc., are downregulated, and anti-apoptotic factors like Bcl2, Bcl-XL, caspase inhibitors, etc., are overexpressed (Evan and Vousden, 2001; Hassan et al., 2014; Kaczanowski, 2016). Unfortunately, various chemotherapeutic agents fail because of loss of p53 function or due to overexpression of the antiapoptotic proteins. Moreover, cancer cells may employ various mechanisms to escape effective treatment, leading to recurrence (Sabnis and Bivona, 2019). Consequently, there is a constant need to develop better counter-strategies to fight the disease efficiently.

Despite major differences between cancer types, most of the cancerous growths acquire some common features that can be used as targets to induce cancer-specific cell death. These include their ability to evade apoptosis; metabolic reprogramming; self-sufficiency in growth signals; ability to invade distant tissues/organs and metastasize; ability to evade immune surveillance; metabolic, mitotic, oxidative, DNA damage, and proteotoxic stresses; sustained angiogenesis, and insensitivity to anti-growth signals and consequent limitless replication (Hanahan and Weinberg, 2000; Kroemer and Pouyssegur, 2008; Luo et al., 2009; Ward and Thompson, 2012). Development of cancer-specific treatment regimens with no or minimum side-effects is the cornerstone of the modern cancer research globally which includes identification of novel vulnerabilities in cancer cells and targeted therapeutics such as anti-angiogenic agents, immunomodulators, monoclonal antibodies, nanoparticles, irreversible electroporation, thermal ablation, CRISPR/Cas9-based gene editing, exosome vehicles, etc. (Amin and Hammers, 2018; Andreozzi et al., 2018; Bouvry et al., 2018; Dar et al., 2018; Darrason et al., 2018; Goedegebuure et al., 2018; Kozyrakis et al., 2018; Karimian et al., 2019; Kohnken and Mishra, 2019; Pacheco and Bunn, 2019; Wu et al., 2019; Xie et al., 2019; Zhang et al., 2019). These strategies can be used in combination with conventional treatment strategies to further ameliorate the health status of humans as well as other animal species.

## Viruses and Their Genes as Anti-Cancer (Oncolytic) Agents

Viruses have the innate ability to infect particular types of tissues by attaching to specific receptors present on the cell surface. Due to this ability called tropism, viruses can be used to target tumors of such specific tissues. They can be considered as nano-delivery vehicles due to their small size, while their genomes can be thought of as oncolytic drugs. Viruses can be exploited through genetic modification to develop treatment regimens to achieve efficient tumor targeting and minimal toxicities, as they employ multiple mechanisms that can restrict their activity to cancer-affected tissues (Pavet et al., 2011; LaRocca and Warner, 2018; Liu and Guo, 2018; Pol et al., 2018; Raja et al., 2018). The first oncolytic virus to be approved by a state regulator is a genetically modified adenovirus named “H101,” marketed under the trade name Oncorine (Liang, 2018). Another virus ECHO-7 belonging to family *Echovirus* was approved in Latvia in 2004 and is marketed under the name Rigvir. It supposedly possesses immuno-activating and oncolytic properties, although the beneficial effects of Rigvir have been a subject of debate (Doniņa et al., 2015; Alberts et al., 2018; Tilgase et al., 2018). Other examples of oncolytic viruses at different stages of research include Herpes Simplex viruses, Newcastle Disease Virus, Vesicular Stomatitis Virus, Adenoviruses, Reovirus, Parvoviruses, Measles Virus, Vaccinia Virus, Rabies Virus, Poliovirus, etc. (Ravindra et al., 2008; Angelova et al., 2009; Raykov et al., 2009; Singh et al., 2012; Goldufsky et al., 2013; Niemann and Kuhnel, 2017; Desjardins et al., 2018). However, using viruses as therapeutic agents poses various risks, which include eliciting host immune reaction, causing toxicities, dampening effect on subsequent administration, narrow therapeutic indices, damage to normal cells that may express the interacting receptor, and socio-environmental hazards due to viral re-emergence (Fountzilas et al., 2017).

To avoid the side-effects associated with using whole viruses as oncolytic agents, oncolytic viral gene therapy instead employs a single viral gene (or a combination of genes) which on ectopic expression finds and selectively destroys malignant cells. Oncolytic genes are non-toxic and biodegradable, have a large therapeutic index, have a limited pathogenicity to normal tissue, can be repeatedly administered without loss of function, do not pose serious socio-environmental hazards, escape immune system unlike complete viral particles and can be effectively targeted using peptide vehicles (like peptide nano-cages) to induce apoptosis in transformed cells (Noteborn, 2009; Pavet et al., 2011; Backendorf and Noteborn, 2014; Gupta et al., 2015; Lezhnin et al., 2015).

## Apoptin as an Oncolytic Agent

Chicken Anemia Virus (CAV) is a member of genus *Gyrovirus* and family *Anelloviridae*, having a 2.3 kb long circular ssDNA genome which replicates through a dsDNA intermediate. The genome codes for a polycistronic polyadenylated mRNA consisting of three overlapping open reading frames (ORFs) for VP1 (51.6 kDa), VP2 (24.0 kDa), and VP3 (13.6 kDa). CAV transiently causes severe anemia and immunodeficiency due to the destruction of erythroblastoid cells and depletion of cortical thymocytes, respectively, in young chickens. It also causes cytopathogenic effects in chicken thymocytes and cultured transformed mononuclear cells. It has been observed that VP3, also known as Apoptin, accumulates in nucleus as fine granules earlier in infection and larger aggregates later to induce apoptosis in chicken mononuclear cells via DNA fragmentation and condensation (Noteborn and Koch, 1995).

Apoptin is a proline-rich protein capable of inducing apoptosis in cancer cells in a selective manner (Noteborn and Koch, 1995; Singh et al., 2015). This activity was reported for the first time by Zhuang et al. (1995a,c) in human osteosarcoma cells and other human hematological tumor cell-lines. Apoptin has no toxic or transforming activity in normal lymphoid, dermal, fibroblast, epidermal, endothelial, and smooth muscle cells (Danen-van Oorschot et al., 1997b, 1999b). In normal cells, Apoptin is found predominantly in the cytoplasm, whereas in transformed (even if non-tumorigenic) and malignant cells, it is located in the nucleus and able to induce apoptosis (Zhuang et al., 1995a; Danen-van Oorschot et al., 1997a, 2003; Rollano Penaloza et al., 2014; Liu et al., 2016). It has been found to be able to kill transformed cells irrespective of their p53, Bcl2, and/or BCR-ABL1 expression (Zhuang et al., 1995a,b,c; Danen-van Oorschot et al., 1997b). Apoptin induced apoptosis in cancer cells is mitochondrial-mediated caspase-3-dependent, whereby the time point of caspase-3 activation varies from one cell type to another (Danen-van Oorschot et al., 2000; Schoop et al., 2004; Maddika et al., 2005, 2006; Burek et al., 2006; Yuan et al., 2013b). Mitotic catastrophe is another mechanism through which Apoptin has been reported to induce cell death in human osteosarcoma cells (Lanz et al., 2013).

## Structural Domains

The biophysical attributes of the protein have been discussed in detail by Castro et al. (2018) in their review. Apoptin has little secondary, and almost no tertiary or quaternary structures, *in vivo* or *in vitro*, and is expressed in the cytoplasm in a filamentous form (Leliveld et al., 2003c). Each Apoptin monomer contains two different domains, one at each end.

### C-Terminal Domain (AA74–121)

Also called tumor cell enhanced nuclear targeting site (tNTS), it is responsible for nuclear localization of Apoptin in cancer cells, and has ∼20% of pro-apoptotic activity (Poon et al., 2005a, b; Kuusisto et al., 2008; Maddika et al., 2009; Nastasie et al., 2015). It is made up of following subdomains (Danen-van Oorschot et al., 2003; Poon et al., 2005a, b).

#### Nuclear Localization Signal (NLS) (Danen-van Oorschot et al., 2003)

The nuclear localization signal (NLS) is bipartite in nature, spanning from amino acids 82 to 88 (NLS1) and 111 to 121 (NLS2). These sequences drive Apoptin import into the nucleus in normal as well as cancer cells.

#### Nuclear Export Signal (NES) (AA97–105) (Poon et al., 2005a)

This sequence is recognized by exportin-1 (CRM-1), and drives the Apoptin transport from the nucleus out into the cytoplasm. It is active only in normal cells due to the inhibitory effect of phosphorylation at T108 in cancer cells.

#### Tumor Cell-Specific Phosphorylation Site (T108) (Rohn et al., 2002, 2005; Wadia et al., 2004)

Apoptin is phosphorylated at Thr108 *in vivo* as well as *in vitro* robustly in tumor cells and negligibly in normal cells by a cancer cell-specific kinase. This phosphorylation inhibits nuclear export of Apoptin while the nuclear import is maintained, thereby resulting in its nuclear accumulation in cancer cells (Poon et al., 2005a).

### N-Terminal Domain (AA1–73)

In addition to the C-terminal domain, the N-terminal domain also mediates some of the apoptotic pathways (Danen-van Oorschot et al., 2003). This domain has the following sub-domains:

#### Multimerization Center (Leliveld et al., 2003c)

It spans amino acid residues 29–69, and is involved in spontaneous multimerization of Apoptin to form globular multimers that bind DNA. The flanking amino acids of a putative amphipathic β-hairpin (AA32–46) in this region determine optimal multimerization.

#### Nuclear Retention Signal (NRS) (Poon et al., 2005b)

This leucine-rich tract spans amino acids 33–46. It facilitates the nuclear accumulation of Apoptin in the presence of bipartite NLS.

## Mechanism of Action

**FIGURE 1:**
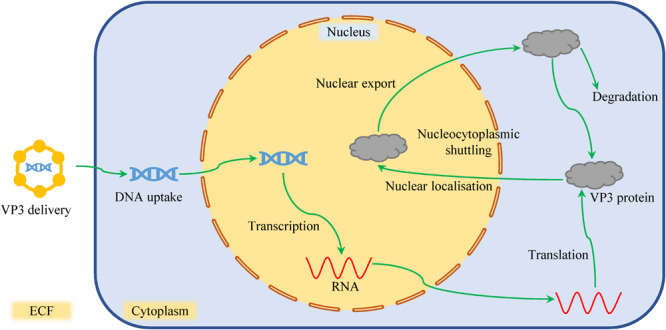
The sojourn of VP3 in normal cells. After the VP3 gene has been delivered into a normal cell, it undergoes transcription and translation. However, due to lack of cancer-specific kinases, the protein shuttles between the cytoplasm and the nucleus without being activated. The filamentous Apoptin becomes aggregated toward the cell margins, epitope-shielded, and eventually degraded by proteasomes without harming the cells.

The N- and C-terminal domains and different combinations of their sub-domains have been reported to bind DNA and induce apoptosis independently to various extents (Danen-van Oorschot et al., 2003; Heckl et al., 2008; Yang et al., 2012; Shen et al., 2013; Song et al., 2016; Ruiz-Martinez et al., 2017a; Wang et al., 2017; Zhang et al., 2017a). In normal cells, the filamentous Apoptin becomes aggregated toward the cell margins, epitope-shielded and eventually degraded by proteasomes without harming the cells (Zhang et al., 2003; Rohn and Noteborn, 2004; Lanz et al., 2012) as shown in Figure 1. The apoptosis induced by the entire protein in cancer cells correlates with its nuclear localization and multimerization, as translocation to elsewhere in the cell results in the complete abolition of activity (Guelen et al., 2004). However, nuclear localization is not sufficient to induce cell death as forcing Apoptin into the nucleus of normal cells does not result in apoptosis, which points toward additional pathways activated or cellular environmental conditions prevailing within a cancer cell (Rohn et al., 2002; Danen-van Oorschot et al., 2003). To induce apoptosis, a threshold level of intracellular Apoptin must be reached after it has been activated (Guelen et al., 2004).

**FIGURE 2:**
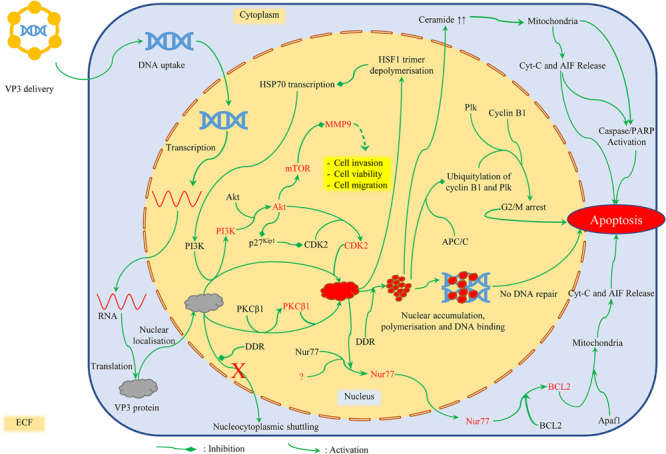
Mechanism of cancer cell death induced by Apoptin. DNA damage response (DDR) and T108 phosphorylation promote nuclear localization of Apoptin. Once phosphorylated, activated Apoptin starts to accumulate within the nucleus. Nuclear localization and accumulation of Apoptin thus prevent the dividing cancer cells from repairing their DNA lesions, forcing them to undergo apoptosis. Apoptin also promotes depolymerization of HSF1 trimers and inhibits HSP70 transcription. This in turn downregulates MMP-9 expression via RTK/PI3K/Akt/mTOR reducing cell viability, migration, and invasion. It also induces apoptosis via mitochondrial pathway (by activating Nur77 and Bcl2 or Cyt-C and AIF release), or through G2/M checkpoint arrest (in association with Plk and Cyclin B1). Features in the image are not to scale. Red font color depicts activation of the protein mentioned.

Kucharski et al. (2011, 2016) proposed that Apoptin activation in cancer cells is a two-step process: In the first step, DNA damage response (DDR), which is constitutively activated in most cancer cell lines, promotes nuclear localization of Apoptin. In primary cells with induced DDR signaling, nuclear localization of Apoptin has been reported to inhibit the formation of p53-binding protein 1 (53BP1)-containing DNA damage foci. This is achieved by sequestration and degradation of Mediator of DNA damage checkpoint protein 1 (MDC1). In the second step, a nuclear kinase phosphorylates Apoptin at T108, thus promoting its nuclear accumulation. Nuclear localization and accumulation of Apoptin thus prevent the dividing cancer cells from repairing their DNA lesions, forcing them to undergo apoptosis (Kucharski et al., 2011). Figure 2 provides an outline of Apoptin induced cell death within a cancer cell (details given below).

Once Apoptin has been activated and nuclear accumulation has begun, about 20–40 Apoptin subunits undergo spontaneous irreversible semi-random aggregation to form multimers that subsequently form protein-DNA superstructures (Leliveld et al., 2003a, c, 2004; Guelen et al., 2004). The multimer has a highly stable core of non-exchangeable subunits attached through hydrophobic interactions to exchangeable subunits (Leliveld et al., 2003b; Ruiz-Martinez et al., 2017b). Each bacterially expressed Apoptin-DNA complex is 200 nm in size and contains 20 Apoptin monomers and ∼3.8 kb of dsDNA (Leliveld et al., 2003a, b; Guelen et al., 2004). One Apoptin multimer contains eight independent non-specific DNA-binding sites which preferentially bind strand ends and interfere with DNA transcription, synthesis, and repair to induce cell death (Leliveld et al., 2003a). Apoptin multimers have been found to be localized in DNA-dense nucleoli, heterochromatin, and promyelocytic leukemia protein nuclear bodies (PML-NBs) (Leliveld et al., 2003a; Heilman et al., 2006; Janssen et al., 2007). In PML-NBs, Apoptin associates with sequestered APC/C complexes to induce apoptosis; however, efficient apoptosis is also induced in reduced or no PML expressing cells (Heilman et al., 2006; Janssen et al., 2007).

Apoptin induced apoptosis is also regulated by activation of Apoptotic protease activating factor 1 (Apaf-1) apoptosome through Bcl-2 (Burek et al., 2006). As a result, an apoptosome-dependent and Bcl-2-modulated death pathway is activated, followed by the release of cytochrome-c (Cyt-c) and Apoptosis-inducing factor (AIF) from mitochondria (Maddika et al., 2005). Cyt-c release is preceded by Apoptin-induced elevation of tumor suppressor lipid ceramide, and followed by activation of caspase 3 and execution of apoptosis (Liu et al., 2006a, b). In dividing osteosarcoma cells, Apoptin expression causes abnormal spindle formation, followed by cell cycle arrest in a p53-independent manner. The cells cannot progress through anaphase and telophase and undergo apoptosis either during mitosis or during the interphase following the delayed mitosis (mitotic catastrophe) (Lanz et al., 2013). In addition to killing cells by preventing DNA repair and inducing mitotic catastrophe, Apoptin induces faster and more efficient cytotoxicity in epithelioid carcinoma xenografts when delivered using a recombinant vaccinia virus. This mechanism involves changes in cytoskeleton whereby tumor tissue is replaced by a filamentous material (Kochneva et al., 2014).However, involvement of alternate pathways of Apoptin activation and consequent induction of apoptosis have been reported. Some of these are discussed below.

### Nature of Cancer Cell-Specific Kinase

It has been a matter of debate whether it is the DDR or a cancer cell-specific kinase that limits the activity of Apoptin to cancer cells. While many reports suggest that cancer cells differentially express a kinase that phosphorylates Apoptin at T108 thereby activating it, the nature and identity of such a kinase have remained an enigma. Protein kinase C (PKC), cyclin-dependent kinases (CDKs) 1 and 2, homeodomain-interacting protein kinase 2 (HIPK2), phosphoinositide 3-kinase (PI3K), and checkpoint kinases (Chk) are some of the kinases that have been incriminated in Apoptin activation (Los et al., 2009; Maddika et al., 2009; Kucharski et al., 2011, 2016; Zhao et al., 2013; Bullenkamp et al., 2015).

When expressed in cancer cells, Apoptin has been reported to interact with and activate PKCβ1. Once activated, PKC phosphorylates Apoptin, which inactivates nuclear export signal (NES) and drives Apoptin accumulation within the nucleus (Bullenkamp et al., 2015). On similar lines, Apoptin has been reported to interact with PI3K. PI3K is constitutively activated which in turn activates Akt and causes it to accumulate within the nucleus. Once in the nucleus, Akt activates cyclin A-associated CDK2 by direct phosphorylation, and by inducing degradation of its inhibitor p27^Kip1^. Once activated, CDK2 phosphorylates Apoptin, activates it, and causes it to accumulate within the nucleus (Los et al., 2009; Maddika et al., 2009). Akt phosphorylation has also been reported to occur in recombinant Apoptin (rApoptin)-induced apoptosis (Hou et al., 2018). Similarly, it has been observed that CDK1 is active in hepatocellular carcinoma cells, where its knockdown affects Apoptin localization and activity, thus pointing toward an interaction between the two proteins, and possibly CDK1-driven Apoptin phosphorylation (Zhao et al., 2013).

### Site of Apoptin Phosphorylation and Activation

T108 is not the only site that can be phosphorylated by cancer cell-specific kinases to drive nuclear accumulation of Apoptin. In the case of a mutation in T108, the adjacent T107 becomes opportunistically phosphorylated, as a result maintaining the cancer cell-specific activity of the protein (Danen-van Oorschot et al., 2003; Guelen et al., 2004; Rohn et al., 2005; Lee Y. H. et al., 2007). However, the existence of phosphorylation sites elsewhere in the protein which perform the same function has been suggested as well. Apparently, T56/T61 are the other residues that are believed to play a role. This belief is strengthened by the facts that both the residues are located within Checkpoint kinase (Chk1/2) consensus sites, that a loss of function of these residues prevents nuclear accumulation of Apoptin and that Chk2 silencing rescues cancer cells from Apoptin-induced cell death (Kucharski et al., 2011).

### Role of Bcl-2 in Apoptin-Induced Apoptosis

An important protein whose role in Apoptin-induced apoptosis has been debated is the pro-survival B-cell lymphoma 2 (Bcl-2) protein. Co-expression of BCL-2 and Apoptin has been reported to enhance the apoptotic activity of Apoptin to various extents in different cancer cell types, but not in normal cells (Danen-van Oorschot et al., 1997a, b, 1999b; Burek et al., 2006). Nur77 is a steroid receptor whose interaction with Apoptin in the nucleus of some cells might explain the debatable role of Bcl2 in Apoptin-induced apoptosis (Los et al., 2009). Phosphorylation of Nur77 has been detected in cells treated with rApoptin. Subsequently, Nur-77 is translocated into the cytoplasm, thus transmitting the Apoptin-induced pro-apoptotic signal to the mitochondria (Hou et al., 2018). Once in the cytoplasm, it interacts with Bcl-2 and converts it from a pro-survival protein to a pro-apoptotic one.

### Role of Caspases and Heat Shock Proteins (HSPs)

Cleavage of caspase-3, caspase-4, caspase-7, caspase-9, caspase-12, and PARP, but not caspase-8, has been observed. This means that Apoptin triggers apoptosis via classical mitochondrial/intrinsic pathway, independent of the death receptor/extrinsic pathway (Danen-van Oorschot et al., 2000; Maddika et al., 2005; Burek et al., 2006; Singh et al., 2015; Bae et al., 2017a). Also, Apoptin activity is delayed or inhibited by some caspase inhibitors (e.g., Baculovirus p35; Z-VAD-FMK) but not by others (e.g., Cowpox virus CrmA), which implies that activation of only a few caspases like caspase-3 and other downstream caspases is sufficient to induce Apoptin activity (Danen-van Oorschot et al., 2000; Pietersen et al., 2004; Burek et al., 2006). Moreover, Apoptin promotes depolymerization of HSF1 trimers and competes with HSF1 binding to Heat Shock Element (HSE) in the promoter of heat shock protein (HSP)70. This binding achieved via Apoptin’s bipartite NLS, and enhanced by leucine-rich sequence between NLS1 and NLS2, inhibits HSP70 transcription, downregulates it, and induces apoptosis as a consequence (Yuan et al., 2013a; Zhang et al., 2017a, b). An Apoptin derived peptide (ADP) has also been found to interact with SH3 domain on the HSE of HSP70 promoter, followed by RTK/PI3K/Akt/mTOR (mammalian target of rapamycin) signaling which downregulates MMP-9 expression. As a result, there is a reduction in cell viability, migration, and invasion in glioma cells (Song et al., 2016).

### Role of p73 in Apoptin-Induced Apoptosis in p53^–/–^ Cells

One of the advantages of Apoptin-based gene therapy is its ability to induce apoptosis in transformed cells irrespective of the functional status of p53 gene. Proteins like Adenoviral E1B-55K, Adenoviral E1B-21kD, short splice variant of FLICE (FADD-like IL-1beta-converting enzyme)-inhibitory protein (FLIP_S_), X-linked inhibitor of apoptosis protein (XIAP), Bcl-2-associated anthanogene 1 (BAG1), Bcl-XL, etc., which inhibit p53-induced apoptotic pathway, do not block Apoptin-induced apoptosis or do so in a cell type-specific manner, which demonstrates the p53-independent cytotoxicity of Apoptin (Zhuang et al., 1995c; Danen-van Oorschot et al., 1997a; Schoop et al., 2004; Burek et al., 2006; Liu et al., 2016).

In p53-null transformed cells, Apoptin associates through its C-terminal with Anaphase Promoting Complex Subunit 1 (APC1 subunit) of the Anaphase-Promoting Complex/Cyclosome (APC/C). This association inhibits the ubiquitination and subsequent APC-dependent proteolysis of cyclin B1 and Polo-like kinase (Plk). As a result, these cells undergo G2/M arrest 12h onward and subsequent apoptosis (Teodoro et al., 2004). G2/M arrest followed by apoptosis has also been reported in gastric cancer cells post adenoviral-mediated Apoptin delivery (Li et al., 2013).

When p53 is non-functional, Apoptin induces cell death via p73 pathway. p73 is a protein similar to p53 and occurs in two forms. The transactivation competent (TA) tumor suppressor isoforms are involved in the regulation of cell cycle and apoptosis, whereas NH2-terminally truncated (ΔN) putative oncogenic isoforms are anti-apoptotic in nature. Exogenous expression of TAp73 isoforms has been found to sensitize p53-deficient cancer cells to killing by Apoptin, while ΔNp73α isoform blocks this activity. Increased levels of endogenous p73 have been detected as early as 6 h after Apoptin delivery into various cell lines (Klanrit et al., 2008).

### Apoptin’s Interaction With Other Proteins Might Affect Its Cancer Cell Specificity

Once inside the cell, Apoptin interacts with several proteins. HSPA9 is an anti-apoptotic and cellular anti-aging member of the Hsp70 family. When overexpressed, it restricts Apoptin partially to the cytoplasm, thus affecting its cancer-specific apoptotic potential (Peng et al., 2013). When co-expressed with members of the death domain superfamily, Apoptin colocalizes with CARD containing protein Bcl10 and DD-containing protein FADD (Guelen et al., 2004). Apoptin also interacts with human Death Effector Domain Associated Factor (DEDAF), whose overexpression has been seen to inhibit anaplastic thyroid cancer (ATC) cell proliferation, invasion, and cisplatin resistance (Danen-van Oorschot et al., 2004; Tong et al., 2019). The possibility that the pathways, through which each exhibits its action, intersect at some point cannot be ruled out (Danen-van Oorschot et al., 2004). In chronic myeloid leukemia (CML) cells, Apoptin interacts through a proline-rich segment with the SH3 domain of BCR-ABL-1. This interaction inhibits BCR-ABL-1 kinase activity and regulates the activity of its downstream targets (Panigrahi et al., 2012; Jangamreddy et al., 2014). However, Apoptin in functional in CML cells irrespective of the functional status of BCR-ABL-1, which may be attributed to its multiple interaction partners within the cells (Jangamreddy et al., 2014).

As stated earlier, Apoptin induces apoptosis in cancer cells in a selective manner without affecting the normal cells. Fast proliferating cancer cells are more susceptible to and show quick Apoptin-induced apoptosis. However, Apoptin can also induce apoptosis in non-dividing cancer cells arrested in the G0/G1 phase by caspase-3 activation (Danen-van Oorschot et al., 2000; Lanz et al., 2013). The differential cell killing activity of the protein has been attributed to various factors, but the complete mechanism is unknown. The fact that most of the Apoptin’s protein partners like DEDAF, Hippi, Akt1, APC1, Nur77, HSC70, etc., are expressed only in tumor cells, and that Apoptin inhibits HSP70 transcription in these cells might explain this activity to some extent (Guelen et al., 2004; Zhang et al., 2017a). In the normal human HEL cells, Apoptin has been found to colocalize with Hippi in the cytoplasm; whereas in cancerous (HeLa) cells, they show only modest co-localization. The inability of Apoptin to enter the nucleus after it binds to Hippi in normal cells may hinder its activity, and render normal cells less susceptible to apoptosis via Hip-1-Hippi-caspase-8 pathway (Cheng et al., 2003). Differential expression of cancer cell-specific kinases which activate Apoptin through phosphorylation may also explain the selective apoptosis it induces. Another explanation can be deduced from the fact that Apoptin on interaction with Bcl-2 and Akt completely hijacks their function and reverses their role as pro-survival factors in cancer cells.

## Delivery Systems

Many anti-cancer agents need a functional p53 to elicit their activity, which is often compromised in cancer cells. The p53-independence of Apoptin induced apoptosis makes Apoptin an interesting therapeutic choice. Another factor that determines the suitability of a molecule as a drug is its safety toward the normal cells and tissues. The ability of Apoptin to induce apoptosis selectively in cancer cells renders it safe as a therapeutic agent. However, additional measures can always be deployed to achieve safer oncolysis. This can be ensured by choosing a suitable delivery vehicle. Since the activity of Apoptin protein is concentration dependent, the delivery vehicle must additionally ensure an optimum level of protein expression. Various delivery systems have been employed to achieve gene therapy in cancerous or other cells (El-Aneed, 2004; Mali, 2013). Several delivery methods have been employed to deliver Apoptin. Rollano Penaloza et al. (2014) and Castro et al. (2018) have provided a detailed account in their respective reviews. Some of the methods are briefly mentioned below.

### Viral Vectors

The small size of the gene coding for Apoptin ensures that it can be delivered with viral vectors with low insertional capacities. Adenoviral-mediated delivery of Apoptin to various cholangiocarcinoma cell lines causes complete eradication of cells, irrespective of their oncogenic mutations and chemotherapeutic resistance (Pietersen et al., 2004). Similar results have been obtained in human hepatomas grown as subcutaneous xenografts in immune-deficient mice. Intra-tumoral adenoviral delivery of Apoptin resulted in complete tumor regression and increased long-term survival of these animals (van der Eb et al., 2002). Also, adenoviral delivery of Apoptin with a human Telomerase reverse transcriptase (hTERT) promoter or SV40 promoter has been reported to have enhanced cytotoxicity, inhibit the migration and invasion of cancer cells *in vitro*, and prolong survival of mice with xenograft tumors *in vivo* (Song, 2005; Cui et al., 2019; Tian et al., 2020). In addition to direct delivery to cancer cell lines and to xenografts of human tumors in mice through intra-tumoral route, effective Apoptin delivery through adenovirus has been achieved via intraperitoneal, subcutaneous, or intravenous injections against xenogeneic tumors in mice (Pietersen et al., 1999). Boulaiz et al. (2014) have used a retroviral-mediated conditional gene expression system to deliver Apoptin to a colon cancer cell line. A recombinant vaccinia virus expressing Apoptin has been reported to involve cytoskeletal changes which result in the replacement of epithelioid carcinoma xenograft tissue with a filamentous material (Kochneva et al., 2014). Lee J. J. et al. (2007) reported that Apoptin delivered using lentiviral vectors is more efficient at inducing apoptosis in canine mammary tumor (CMT) cells as compared to lipofection.

### Plasmid Vectors

Viral vectors often run the risk of eliciting an immune response, which reduces their efficacy on repeated administration. Non-viral vectors like plasmids are non-immunogenic, can accommodate a larger construct, and allow for repeated administrations without attenuating the effect (Mali, 2013). DNA loaded onto a plasmid can be transfected into mammalian cell lines either in a naked form, as a projectile (gene gun), through electroporation or as a payload within a liposome (lipofection). Apoptin delivered to cholangiocarcinoma cell lines has been reported to cause extensive apoptosis in these cells (Pietersen et al., 2004). Similar results have been reported from experiments on, but not limited to, mouse mammary tumor cells, human cervical cancer cell line, prostate carcinoma cells, and more recently, clinically occurring cases of canine transmissible venereal tumor (CTVT; Singh et al., 2015; Gupta et al., 2016; Bhat et al., 2017; Mohammadi et al., 2017). In electroporation, an electric field is applied to the cells to enhance the uptake of nucleic acids. However, this method is not as effective as others. Electroporation-mediated delivery of Apoptin has been shown to induce only a transient effect on tumor growth in murine melanoma and renal carcinoma models (Mitrus et al., 2005).

### Nanoparticle-Based Delivery Systems

Nanoparticle-based delivery systems called nanocarriers, usually < 100 nm in size, can sequester a drug at its site of action and avoid side-effects that may arise due to its distribution throughout the body. This differential biodistribution of nanoparticles is of considerable importance in cancer therapy, where it can maximize efficacy and minimize toxicities (Wang et al., 2012). Yang et al. (2020) recently synthesized nanocomplexes of fluorinated branched polyethyleneimine and a modified Apoptin plasmid construct. The Apoptin-plasmid construct was modified to deliver Apoptin to the neighboring cells after synthesis in the transfected cells. The complexes were reported to have efficiently transfected into, and caused apoptosis in, non-small cell lung carcinoma cells *in vitro* as well as *in vivo* (Yang et al., 2020).

Repebodies are non-immunoglobulin antibodies composed of leucine-rich repeat modules engineered based on similar molecules found in the adaptive immune system in jawless vertebrates, which can be used in targeted drug delivery (Lee et al., 2012). Repebody-drug conjugates have been found to have higher stability, minimal off-targets, and remarkable anti-tumor activity *in vivo* (Lee et al., 2015). Lee et al. (2017) designed repebody-Apoptin conjugates, which can be delivered as a systemic injectable preparation. These self-assembling fusion protein nanoparticles show anti-tumor activity in human breast cancer (hBC) xenografts in mice through cooperative action (Lee et al., 2017).

Viral and bacteriophage particles represent nature’s own nanoparticles. Differential biodistribution is achieved by their tropism—the ability to infect and multiply in specific cell types. Phage particles have an outer coat that has evolved to protect their genome inside. Phage particles can be engineered, or a gene of interest can be inserted into the phage genome and its protein will be expressed on the phage surface. These properties can be exploited to achieve targeted delivery of oncolytic gene cassettes against cancer (Hosseinidoust, 2017; Petrenko and Gillespie, 2017). Shoae-Hassani et al. (2013) designed Apoptin expressing λ Phage nanobioparticles and observed significant inhibition of growth in hBC cells *in vitro* and suppression of xenograft tumor growth *in vivo* in mice. Bacterial magnetic particles (BMPs) are membrane-bound, nanoscale (40–120 nm) sized biocompatible Fe_3_O_4_ particles found in magnetotactic bacteria that can be used as a gene delivery system (Wang et al., 2018). Wang et al. (2018) have recently used BMPs to deliver Apoptin-cecropin B [an antibacterial peptide (ABP)] containing plasmids into human hepatoma cells and reported superiority of BMPs as delivery agents over lipofection for cancer gene therapy.

### Peptide and Protein Vehicles

Cell-penetrating peptides (CPPs) comprise other class of delivery agents that can be used to efficiently deliver drugs as cargo into the cells (Milletti, 2012). HIV trans-activating transcriptional (TAT) protein was the first CPP to be discovered independently by two groups in 1998 (Kylie and David, 2006). Guelen et al. (2004) used HIV-TAT to deliver Apoptin protein to various normal and cancer cells and found that cancer cell-specific cytotoxicity of Apoptin was still maintained. TAT-Apoptin conjugates have also been found to be effective against bladder cancer cells and hepatocellular carcinoma xenografts in nude mice (Li et al., 2012; Ma et al., 2012). Zhou et al. designed a CPP CPP-MT23 with the ability to efficiently target and penetrate mouse melanoma cells. MT23-Apoptin fusion proteins were found to induce apoptosis in mouse melanoma cells *in vitro* and inhibit mouse melanoma tumor growth *in vivo* (Zhou et al., 2017). Wang et al. (2016) used *in silico* approach to design a CPP from human C-terminal fragment of lysine-specific demethylase 4A (KDM4A). Since this peptide, called hPP10, is a human-derived peptide, there are decreased chances of eliciting an immune response. This peptide can deliver Apoptin to cancer cells and induce cancer cell-specific apoptosis (Wang et al., 2016). Another CPP, called PTD-4, has been reported to show nuclear localization in human cervical carcinoma (HeLa) cells, gastric tumor derived cells, and human hepatocarcinoma cells *in vitro* (Sun et al., 2009; Liu et al., 2016). Similarly, significant tumor growth inhibition has been reported in human hepatic, gastric, and cervical carcinoma xenografts in mice (Sun et al., 2009).

Dendrimers are repeatedly branched macromolecules, each consisting of a central multifunctional core, branches arising from the core, and peripheral functionalities. Various types of dendrimers have been designed as carriers for anticancer, anti-inflammatory, and antimicrobial agents to achieve targeted/controlled drug delivery, maximize selectivity, and minimize toxicity (da Silva Santos et al., 2016). Polyamidoamine (PAMAM) dendrimers, whose peripheral functionality consists of numerous active amine groups, were the first to be reported. The particle size can be controlled so as to achieve tissue/cell-specific targeting (Li et al., 2018). Moreover, PAMAM transfection efficiency, safety, and biodegradability can be enhanced by using amino acid-conjugated PAMAM ester derivatives as delivery agents (Nam et al., 2009). An et al. (2013) used PAM-RG4, an arginine derivative of PAMAM, to deliver Apoptin into human glioblastoma cells *in vitro* and brain tumor xenografts in mice. They reported high transfection efficiency, induction of apoptosis in cell cultures, and inhibition of tumor growth *in vivo*. Beneficial effects on hindlimb function and inhibition of tumor growth have been reported when PAM-RG4 was used to deliver VP3 gene to intramedullary spinal cord tumor in a rat model (Pennant et al., 2014). In addition to mono-amino acid conjugates, di- and tripeptide conjugates of PAMAMs have also been used to effectively deliver Apoptin into human primary glioma cells, human hepatocellular carcinoma cells, and glioblastoma cells (Bae et al., 2016, 2017a,b, 2019).

Due to the exceptionally high nutritional requirements of cancer cells, human serum albumin (HSA), an important source of nutrition, accumulates in solid tumors. This tendency of HSA can be exploited to turn HSA into a tumor targeting delivery agent. Wu et al. (2017) designed a plasmid construct with Apoptin and HSA genes, and studied its tumor specific cytotoxicity in breast cancer cells. Significant negative effects on cell viability *in vitro* and on tumor growth *in vivo* in a mouse mammary tumor model were observed. The construct was able to induce apoptosis in tumor cells without affecting normal cells (Wu et al., 2017).

### Mesenchymal Stem Cell (MSC) Delivery

Gazit et al. (1999) reported the use of engineered mesenchymal stem cells (MSCs) as gene delivery as agents. Since then, MSCs have generated considerable interest in case of various diseases. They have prominently been used to deliver, and achieve local secretion of various therapeutic proteins, prodrug activators, and oncolytic viruses against cancer, as covered in their review by Dwyer et al. (2010). As far as Apoptin is concerned, conditional activation of MSC-delivered Apoptin has been attempted which resulted in induction of apoptosis and inhibition of tumor growth, respectively, in liver cancer cells *in vitro*, and hepatic and lung carcinoma mice models *in vivo* (Du et al., 2015; Zhang et al., 2016).

### Bacteria as Vectors

Just like viruses, the phenomenon called tropism where the pathogen infects and replicates within a specific tissue is also seen in some bacteria. In a narrower context, the bacterium may invade a particular organelle or extracellular space within the tissue (McCall et al., 2016). This property of bacteria can be exploited to achieve targeted drug delivery against various diseases, including gene therapy against cancer. However, the bacterium used should be non-pathogenic or disarmed, and minimally immunogenic. The bacteria are transformed, and the therapeutic gene (bactofection) or its *in situ* translated protein product (alternative gene therapy) is delivered into the eukaryotic cell after infecting the host. Various bacterial species that have been used for this purpose include members of *Listeria*, *Shigella*, *Salmonella*, *Yersinia*, *Agrobacterium*, *Clostridium*, and *Bifidobacterium* genera (Pálffy et al., 2005; Mali, 2013). An attenuated strain of *Salmonella typhimurium* has been used to deliver the VP3 gene into a human laryngeal cancer cell line and a syngeneic nude murine tumor model. The treatment successfully induced apoptosis of cancer cells *in vitro* and delayed tumor growth and reduced tumor vascularity *in vivo* (Guan et al., 2013).

## Combination Therapy

The emergence of resistant cells, that repopulate the tumor and lead to treatment failure, after therapy with a single agent is probably one of the biggest challenges in cancer treatment. The resistance to conventional chemo- and radio-therapies usually arises due to alterations in apoptotic pathways. One of the ways out is to use two or more drugs/strategies simultaneously that target distinct pathways or act through different mechanisms (Bozic et al., 2013). Cells resistant to one drug can be killed by another and *vice versa*. Moreover, additive cytotoxic effects are observed when two or more drugs/strategies are used in combination. In this section, we review the studies that involve combination therapies with Apoptin being one of the agents.

Dacarbazine is the gold standard treatment for malignant melanoma, but the response rates are low and often not sustained. However, administration of PTD4-Apoptin simultaneously with lower dosages of dacarbazine enhanced the cytotoxic effect of dacarbazine against various mouse and human melanoma cell lines *in vitro* and inhibited tumor growth in a mouse melanoma model *in vivo* (Jin et al., 2011). Other conventional anticancer drugs like paclitaxel, etoposide, etc., have been reported to show additive cytotoxicity against various human transformed cell lines (Olijslagers et al., 2007). Radiotherapy, supplemented with VP3 gene therapy, has been found to increase cell death in poorly responding/radioresistant head and neck squamous cell carcinoma cells (Schoop et al., 2010). Cecropin B is an ABP extracted from silkworm which induces pore formation in lipid membranes. ABPs1, a cecropin B-like peptide, given in combination with Apoptin to HeLa cells induces apoptosis. The combination is more effective than either of the two agents used alone, and the cells show membrane pore formation, caspase-3 activation, and upregulation of Bax (Birame et al., 2014). The additive effect of ABPs1 over Apoptin activity is seen in a time- as well as a concentration-dependent manner. Downregulation of Survivin gene was also reported (Birame et al., 2014). Survivin is a caspase-inhibiting, anti-apoptotic protein overexpressed in most of the human cancers, and associated with chemo- and radio-resistance of cancer cells. A combination therapy involving knockdown of Survivin (using miRNA) and simultaneous overexpression of Apoptin with the same vector has been found to be more efficient than either alone (Liu et al., 2008).

Tumor homing peptides (THPs) constitute a class of therapeutic agents that can be targeted to tumor cells which differentially express surface proteins absent in normal cells (Ruoslahti et al., 2010). In addition to being toxic to the cancer cells themselves, they can be used to achieve targeted delivery of other therapeutic agents. The p28 peptide is a THP that enters cancer cells, interacts with the DNA-binding domain of the p53 protein, and induces G2/M arrest. However, the p53 dependence of p28 limits its scope since p53 mutations are widespread within various cancer types. Apoptin being able to act independently of p53 status can thus complement p28 activity against cancer cells. Noei et al. (2018) studied the effect of a p28-Apoptin chimeric protein on breast cancer cells and reported higher toxicity of the fusion protein against various breast cancer cell lines.

Anti-cancer immunotherapy involves the administration of immunostimulants to intensify the immune response against cancer cells, or designed antibodies, fusion proteins, and immunotoxins to specifically target them (Pranchevicius and Vieira, 2013). These agents can be simultaneously administered with other drugs to achieve synergistic action. For example, intra-tumoral administration of Apoptin and Polyinosinic:polycytidylic acid (poly I:C—an immunostimulant) has been reported to inhibit the tumor growth and induce a potent anti-tumor immune response in a mouse mammary tumor model (Gupta et al., 2016). A strong NK-cell activity and tumor specific cytotoxic T lymphocyte (CTL) response against Lewis lung carcinoma (LLC), with significant tumor regression, have also been observed on simultaneous administration of Apoptin and Interleukin-18 (Lian et al., 2007). Similarly, co-expression of Interleukin-24 and Apoptin reduces the growth of and induces apoptosis in hepatic carcinoma cells *in vitro* xenografts in nude mice (Yuan et al., 2013b). Single-chain variable fragments (scFvs) can be used to target cancer-cell-specific antigens with enhanced penetration and rapid clearance. CD22 is a member of immunoglobin superfamily of proteins found on the surface of mature B-cells. It can be used as a therapeutic target antigen in CD22 positive leukemia and lymphoma cells. A fusion product of Apoptin and anti-CD22 scFv combines the cytotoxic effect of Apoptin and targeting capability of anti-CD22 scFv. The protein has been reported to cross the cell membrane and induce apoptosis in B-cell malignancies (Agha Amiri et al., 2017).

In addition to the VP3 gene of CAV, many other organisms also carry oncolytic genes that act via other mechanisms. Since these genes act through independent pathways, they can be used synergistically to induce cancer cell death, with no or low chances of emergence of resistance. For example, in *Escherichia coli*, gef gene codes for a cytotoxic protein that arrests the cell cycle and induces apoptosis in tumor cells. Combined administration of Apoptin and gef kills cancer cells by inducing pore formation, followed by necrosis. The combination has been reported to decrease cell viability, increase necrosis, and induce apoptosis in colon cancer cells more effectively than either of the two genes alone (Boulaiz et al., 2014; Caceres et al., 2019). Melanoma differentiation-associated gene-7 (MDA7) is an anti-tumor gene that has been found to suppress monolayer growth and anchorage dependence of various cancer cell lines, and inhibit tumor growth in tumor xenografts in mice (Mhashilkar et al., 2001). *Salmonella-*mediated combined delivery of MDA7 and VP3 genes has been reported to decrease tumor growth, induce apoptosis, enhance anti-tumor immunity, and inhibit tumor vascularization in a mouse model of gastric sarcoma (Cao et al., 2016). Similarly, NS1 (non-structural protein 1) gene of various species in *Parvovirus* genus exhibits tumor cell specific cytotoxicity through apoptosis, necrosis, cytolysis, and cathepsin-B-mediated death (Gupta et al., 2015). Bhat et al. (2017) reported for the first time partial oncolysis of clinically occurring cases of the CTVT, induced by combined administration of the NS1 gene of *Canine Parvovirus 2* (CPV.NS1) and Apoptin.

## Future Challenges and Prospects

Most of the literature available about Apoptin supports the idea that apoptosis induced by Apoptin is limited to cancer cells. However, there have been reports of Apoptin inducing cell death in normal cells as well (Tavassoli et al., 2005). Although these reports constitute a minuscule, their importance cannot be underestimated. It is of utmost importance to study further the repercussions of VP3 gene therapy in normal cells before it proceeds from preclinical to clinical trials. Apoptin-based gene therapy, at any time in the future, will also have to consider the level of protein expression that is enough to specifically target the cancer cells. This can be achieved by promoter engineering: design promoters optimized to achieve a certain level of protein expression; using promoters that are specifically activated in response to stimuli found in the tumor microenvironment; or using promoters of those genes that are significantly overexpressed in tumor cells. hTERT and Survivin promoter-driven Apoptin expression has been already studied; however, further research is required in this area (Liu et al., 2012; Ye et al., 2013; Qi et al., 2014). Survivin promoter-driven expression of Apoptin in a lentivirus vector leads to tumor cell death, focal necrosis, and tumor growth inhibition in various cell lines *in vitro* as well as tumor xenografts in mice (Ye et al., 2013). Combination therapy is an avenue with ample chances of growth in gene therapy. As mentioned earlier, some naturally occurring viruses are inherently oncotropic, i.e., they infect and replicate in cancer cells, and cause their lysis. However, the oncolytic effect of natural strains is limited and can be enhanced if recombinant virus strains are used. A recombinant H1 parvovirus expressing Apoptin has been reported to induce apoptosis in a wider range of cancer cells than the ones already susceptible to natural strains, without showing additional cytotoxic effects in normal cells (Olijslagers et al., 2001). Similar studies can be replicated with other recombinant viruses expressing Apoptin, or with their genes in combination with Apoptin. However, it remains to be seen to what extent these observations *in vitro* can be replicated faithfully in a clinical setting or an *in vivo* system. Combined administration of CAV.VP3 and CPV.NS1 in CTVT is an interesting example. Apoptin alone was surprisingly found to be more effective at inhibiting tumor growth than the two genes in combination (Bhat et al., 2017). It is the first report of Apoptin being used in a clinical setting, and the results are contrary to expectations. These findings suggest that despite being a potential candidate for gene therapy, it needs considerable effort to take VP3 to a clinical setup. Further, there is scope for an investigation into the delivery of Apoptin to the cancer cells as a protein or gene. For example, the CRISPR/Cas system mediated modification of MSCs *ex vivo*, followed by *in vivo* administration may open the gates for further improvement. The mechanism of Apoptin activation merits further exploration. The identification of nature of one (or more) kinase(s), which we believe act in a tissue- (or cell-) specific manner, that phosphorylates Apoptin will not only reveal the exact mechanism of Apoptin activation but is also important from a clinical point of view. The discovery is expected to have diagnostic value as a biomarker, to identify novel targeting vulnerabilities in cancer cells, and to eventually affect therapeutic decisions.

## Author Contributions

WM and AT conceived the idea for the article and prepared the first draft of the manuscript. WM and RA searched the relevant literature and prepared the figures. RK and SM helped in drafting the manuscript as well as critically revising its contents. All the authors have read and approved the final version of the manuscript, and agreed to be accountable for its contents.

## Conflict of Interest

The authors declare that the research was conducted in the absence of any commercial or financial relationships that could be construed as a potential conflict of interest.
